# Exploring the Importance of Health Literacy for the Quality of Life in Patients with Heart Failure

**DOI:** 10.3390/ijerph15081761

**Published:** 2018-08-16

**Authors:** Marija Jovanić, Marija Zdravković, Dejana Stanisavljević, Aleksandra Jović Vraneš

**Affiliations:** 1 Faculty of Medicine, University of Belgrade, 11 000 Belgrade, Serbia; marija84jovanic@gmail.com; 2 Faculty of Medicine, Clinical Hospital Center Bezanijska kosa, University of Belgrade, 11 000 Belgrade, Serbia; sekcija.kardioloska@gmail.com; 3 Faculty of Medicine, Institute for Medical Statistics and Informatics, University of Belgrade, 11 000 Belgrade, Serbia; sdejana8@yahoo.com; 4 Faculty of Medicine, Institute of Social, Medicine University of Belgrade, 11 000 Belgrade, Serbia

**Keywords:** health literacy, quality of life, public health, heart failure, Minnesota

## Abstract

As with all other chronic noncommunicable diseases, adequate health literacy plays a key role in making the right decisions in the treatment of heart failure. Patients with heart failure and a lower health literacy have a reduced quality of life. A cross-sectional study among 200 patients with heart failure was conducted at a state university hospital in Belgrade, Serbia. The European Health Literacy Questionnaire, HLS-EU-Q47, was used to assess health literacy. Quality of life was measured with the generic SF-36 and the Minnesota Living with Heart Failure Questionnaire. Descriptive and analytical statistical analysis was applied. More than half of the respondents (64%) had limited health literacy. The lowest mean health literacy index (28.01 ± 9.34) was within the disease prevention dimension, where the largest number of respondents showed limited health literacy (70%). Our patients had a poorer quality of life in the physical dimension, and the best scores were identified in the emotional role and social functioning. Health literacy was highly statistically significant and an independent predictor of quality of life (physical, mental, and total quality of life). Improving health literacy can lead to better decisions in the treatment of disease and quality of life in heart failure patients.

## 1. Introduction

Currently, significant effort is being invested in the operation of health systems in an attempt to satisfy the health requirements of patients. Although public health policies should provide certain preconditions for health, individuals also need to take an active role in terms of specific issues and decisions concerning health [1]. The skills required to manage our health in the best possible way also represent the essence of health literacy [2].

Over recent decades, health literacy, considered the key determinant of health, has been receiving increasing research interest [3]. As such, health literacy can be viewed from public health and medical points of view as the: “individual’s knowledge, motivation, and competences to access, understand, appraise, and apply health information in order to make judgements and take decisions in everyday life concerning health care, disease prevention, and health promotion to maintain or improve quality of life during the life course” [4].

However, there is a constant worldwide struggle against chronic noncommunicable diseases. Of all cardiovascular diseases, heart failure represents the leading cause of hospitalization, with over one million people hospitalized per year [5]. Depicted as a pandemic in the literature, heart failure is becoming a global public health problem, affecting approximately 26 million people globally [5,6]. These patients face complex manifestations of symptoms, which require their understanding and a suitable choice of therapy and commitment in order to stabilize the patient’s condition.

As with all other chronic noncommunicable diseases, adequate health literacy plays a key role in making the right decisions in the treatment of heart failure [3]. Low heath literacy is strongly associated with the diminished use of the health system, as well as with poorer outcomes [7]. Previous studies have shown that patients with heart failure and a lower health literacy have a reduced quality of life [6,8,9,10], often much worse than people who suffer from other chronic diseases [11].

Few cross-sectional studies have investigated a direct correlation between health literacy and the quality of life in patients with heart failure [9,12], while several studies [13,14,15,16,17,18] have investigated the impact of health literacy on different self-management interventions and quality of life at home or in clinical settings.

A study carried out on elderly heart failure inpatients showed that health literacy was only associated with the social component of quality of life [12]. Another study using a larger multicentre sample demonstrated that heart failure patients with low health literacy had a worse quality of life, even after adjusting for race, age, health insurance status, and subjective socioeconomic position [9]. This particular study [9] did not specify which components of quality of life were affected.

The abovementioned studies [9,12] used specific questionnaires to evaluate patient quality of life, but this practice has been criticized because it reduces the chances of comparing results between different studies [19,20]. Along with a specific questionnaire, it is recommended that such studies use generic measuring instruments to evaluate the quality of life in order to acquire a more objective picture of the patient’s life, not just disease-related problems [10,20].

Several randomized control studies [13,14,15,16] that examined self-care educational interventions in heart failure patients showed no significant difference between health literacy and quality of life, although DeWalt [16] pointed out that along with a higher level of health literacy, there was a greater improvement in quality of life. Those studies [13,14,15,16,17,18] also used specific instruments for quality of life.

It is very important to consider the problem of health literacy in patients with heart failure from a complete perspective. This is crucial because these patients spend every day of their lives recognizing disease symptoms, which deteriorate their quality of life. However, in order to completely characterize the issues involved, it is necessary to use a measuring instrument that can provide answers to investigations across all dimensions of health literacy [8]. The European questionnaire for the evaluation of health literacy, referred to as “HLS-EU-Q47”, investigates different dimensions of health literacy [3,21] and could therefore be significant in evaluating patients with heart failure, especially if we take into consideration that most previous studies investigating health literacy in heart failure have been conducted in the United States, using the Test of Functional Health Literacy in Adults–Short Form (S-TOFHLA) [8].

Furthermore, the potential influence of sociodemographic variables on the relationship between health literacy and quality of life has not been sufficiently investigated in patients with heart failure [19].

In the present study, our specific research objectives were to:

(1) Assess the health literacy and quality of life of patients treated for heart failure at the Clinical Center “Bežanijska Kosa”,

(2) Identify the factors associated with health literacy, and

(3) Investigate the connection between health literacy and various domains of patients’ quality of life.

## 2. Materials and Methods

### 2.1. Participants

This research was conducted as a cross-sectional study at the Department of Cardiology in the Clinical Center “Bežanijska Kosa”, a state university hospital in Belgrade, Serbia. The study was conducted in accordance with the ethical principles of the Declaration of Helsinki and was approved by the Ethical Committee of the Faculty of Medicine in Belgrade, (reference number: 29/III-6), and by the Ethical Council of the Clinical Center “Bežanijska Kosa” (reference number: 2574/4). The study was conducted between May 2016 and November 2017.

Our specific inclusion criteria were as follows: (1) patients diagnosed with heart failure, mild to moderate impairment of ventricular function, NYHA II and III class (New York Heart Association Classification of Heart Failure) [22] in the stable phase; (2) patients hospitalized for chronic heart failure; and (3) patients older than 18 years who offered to participate on a voluntary basis.

Our exclusion criteria were as follows: untreated malignant diseases; acute serious diseases such as acute cardiac decompensation, acute myocardial infarction, or unstable angina; pulmonary embolism; myocarditis; serious associated diseases; and psychiatric and neurological diseases (with impairments of cognitive function).

### 2.2. Procedure

We interviewed 200 inpatients with heart failure. Each patient completed a questionnaire on the last day of hospitalization when they were in a stable phase. Each patient provided informed written consent, which stated that they would remain anonymous, were participating on a voluntary basis, and that there was no financial arrangement for those participating in the study. All patients were covered by compulsory medical insurance.

### 2.3. Measuring Instruments

In this study, we used four measurement instruments to acquire detailed data of various factors. These instruments are described below.

#### 2.3.1. European Health Literacy Questionnaire (HLS-EU-Q47)

Health literacy was assessed with the European Health Literacy Questionnaire, HLS-EU-Q47, which consists of three groups of questions (health care, disease prevention, and health promotion). These groups included 11–22 questions relating to the availability, understanding, evaluation, and implementation of information relating to health.

Our respondents were required, in accordance with the scientific and research activity of the hospital, to provide complete answers to all questions.

Answers were given on a scale featuring four levels: 1, very difficult; 2, difficult; 3, easy; and 4, very easy. Consequently, the field “I do not know” was specifically excluded. There were no answers in this category.

According to recommendations [23], we scored each questionnaire and performed the following calculation: Index = (mean (per item) − 1) × (50/3).

Health literacy indices were constructed as a general health literacy index with three dimensions: health care, prevention of disease, and health promotion. The highest final score for the health literacy index was 50, while the smallest value was 0.

Health literacy was also categorized in four levels, as “inadequate” (0–25), “problematic” (> 25 to 33), “sufficient” (> 33 to 42), and “excellent” (> 42 to 50). In an attempt to identify vulnerable groups, according to recommendations [21,23], the “inadequate” and “problematic” levels were combined as a single level, which we referred to as “limited health literacy” (0–33).

The European questionnaire has been previously validated, shows good reliability in multinational samples, and is free to use as long as specific recommendations are followed [21,23]. The questionnaire has already been used to test the literacy of specific groups of patients, such as adolescents [3], diabetics [3,24], and patients with heart failure [25]. We used the full version of the questionnaire with a cultural adaptation (linguistic validation) to the Serbian language. The value of Cronbach’s coefficient in our study showed good validity and provided the following results: total index of health literacy *α* = 0.949, health care domain *α* = 0.872, disease prevention *α* = 0.867, and health promotion *α* = 0.888.

#### 2.3.2. Quality of Life Survey Generic Questionnaire (SF-36)

The generic questionnaire for measuring quality of life from the Medical Outcomes Study [26], Short Form 36 Item (SF-36), consists of eight groups of questions related to physical and mental health. Each of these eight groups includes two to 10 questions. Responses to the formulated questions were offered in the form of two-, three-, and five-level scales. The values of two summarized scores were referred to as the physical and mental quality of life, the minimum and maximum values of which were 0 and 100, respectively; the higher the value, the better quality of life [27]. The Serbian version of SF-36 was validated on cardiac patients [27]. In our study, Cronbach’s coefficient was calculated individually for each dimension in order to establish internal consistency [11], and the results were as follows: physical functioning *α* = 0.938, physical role *α* = 0.907, body pain *α* = 0.423, general health *α* = 0.724, vitality *α* = 0.889, social functioning *α* = 0.856, emotional role *α* = 0.926, and mental health *α* = 0.829.

#### 2.3.3. Quality of Life Survey Specific Questionnaire (MLWHF)

A specific questionnaire, the Minnesota Living with Heart Failure Questionnaire (MLWHF) [28], was used to measure quality of life. We used a version of this form which was validated for Serbians [22,29] and featured 21 questions related to the presence of disease symptoms, signs of heart failure disease, disease-caused limitations, staying in hospital for treatment, the cost of treatment, the side effects of drugs, and emotional problems. Answers were offered in the form of six-level scales, from “no” (0) through “very difficult” to “very easy” (5). We evaluated the values of summary scores for physical, mental, and overall quality of life. A total score (minimum and maximum values of 0 and 100, respectively) was calculated by adding up the answers for each question; lower total values referred to a better quality of life. In our study, Cronbach’s coefficient was *α* = 0.908 for the physical dimension, *α* = 0.824 for the emotional dimension, and *α* = 0.928 for the total quality of life.

#### 2.3.4. Patients’ Characteristics and Sociodemographic Questionnaire

An additional questionnaire featuring 14 questions was used to evaluate the sociodemographic characteristics of our respondents. Age was categorized in three categories: up to 50 years, 51–64 years, and older than 65 years. The use of alcohol and cigarettes was evaluated dichotomously. Employment and marital status were categorized dichotomously as employed/unemployed and married/unmarried. Self-perception of financial status and health status was measured with the five-point Likert scale; answers were given on a three-point scale (poor, average, and good), since there were only a few answers.

The number of visits to the doctor was examined on a three-step scale: one to four visits a month, went three months ago, and have not seen a doctor. The level of education was classified into three categories as follows: low (primary school or less than eight years of school completed); medium (secondary school, eight to 12 years of school completed); and high (college or university, more than 12 years of school completed). Blood pressure values were categorized according to specific recommendations [30] as low, normal, or elevated. Body mass index (BMI), according to the recommendation in [31], was calculated using the formula (BMI = kg/m^2^), the values of which were classified into three categories: malnourished (< 18.5), normal (18.5–24.9), or obese (> 25).

### 2.4. Statistical Analysis

Univariate and multivariate analyses were used to investigate descriptive and inferential statistics. Measures of central tendency (mean and median), rate variability (standard deviation) for continuous variables, and absolute frequencies and percentages for categorical variables were used for descriptive statistics. The χ^2^ test (contingency table), t-test, and analysis of variance (ANOVA) were used to test the significance of differences. In addition, linear regression was used to determine independent predictors of health literacy among respondents. Statistical analysis was conducted using the Statistical Package for the Social Sciences (SPSS) version 22.0 (IBM Corp., Armonk, NY, USA). In all cases, a *p* value < 0.05 indicated statistical significance.

## 3. Results

### 3.1. Sociodemographic Characteristics of the Participants in the Study

This study included 200 inpatients with heart failure, surveyed at the Department of Cardiology in the Clinical Center “Bežanijska Kosa”, a state university hospital in Belgrade, Serbia. All patients completed the questionnaires. Table 1 shows the respondents’ sociodemographic characteristics.

The sample contained more male patients, *n* = 119 (59.5%). Respondent age ranged from 36 to 91 years, with a mean of 70.12 ± 9.63 years. The majority of respondents were pensioners, *n* = 172 (86%), who lived in marital union, *n* = 122 (61%).

In terms of educational level, most of the respondents, *n* = 88 (44%), had a medium school diploma. The majority of our respondents, *n* = 173 (86.5%), visited their doctor on a regular basis. More than half of our respondents, *n* = 115 (57%), perceived their health as being bad and every second patient considered their financial status as average.

Only 9% of participants consumed alcohol, and for cigarettes, *n* = 36 (18%). The mean body mass index was 28.17 ± 5.18.

### 3.2. Respondent Health Literacy Level

Assessing health literacy level in heart failure patients, we found that more than half of our respondents had limited health literacy, *n* = 128 (64%). Inadequate and problematic literacy was found in 73 (36.5%) and 55 (27.5%) participants, respectively. Sufficient health literacy was found for *n* = 56 (28%), while only 8% had excellent literacy, as shown in Table 2.

We used the full version of the European Health Literacy Questionnaire, HLS-EU-Q47, where the mean score value of the general health literacy index was 29.23 ± 9.12.

Among the three dimensions, respondents with heart failure showed the lowest mean health literacy index (28.01 ± 9.34) within the disease prevention dimension, which represents the largest number of respondents, *n* = 140 (70%), who showed limited health literacy.

The respondents had the best mean health literacy index (33.03 ± 8.52) in the health care dimension.

### 3.3. Quality of Life of Respondent

In the present study, quality of life in heart failure patients was assessed with two questionnaires, the generic (SF-36) and specific (MLWHF), the results of which are shown in Table 3.

According to the SF-36 questionnaire, our patients with heart failure showed lower mean scores (45.01 ± 24.77) for the physical dimension, while the mental dimension displayed better mean scores (50.35 ± 25.82).

Of all eight dimensions measured with SF-36, the worst values for quality of life were evident in the dimensions of physical role, physical function, vitality, and general health. In addition to this, the best values for quality of life were in the dimensions of body pain, mental health, emotional role, and social function.

Total quality of life score, as measured by the Minnesota questionnaire, was 51.67 ± 24.10. Our patients with heart failure had higher mean scores (24.73 ± 10.87), which indicate a worse physical dimension, while the mental dimension had better mean scores (7.34 ± 6.42).

### 3.4. Factors Associated with Health Literacy Level

The distribution of respondent characteristics based on health literacy levels, as well as on the general health literacy level, is given in Table 4.

We identified, with regard to the sociodemographic features, a higher proportion of heart failure patients with limited health literacy among females (66.7%), between 51 and 64 (66.7%) years old and older than 65 years (63.9%), who were unmarried (70.5%), unemployed (64%), and had completed less than eight years of school (95.7%). Furthermore, limited health literacy was found among those whose perceived financial status and self-perceived health were regarded as poor in 78.4% and 78.3% of patients, respectively. In addition to this, all four participants who were malnourished had limited health literacy.

Significant associations between sociodemographic characteristics and health literacy levels were found for education level (χ^2^ = 110.426; *p* < 0.001), employment status (χ^2^ = 7.461; *p* = 0.024), self-assessment of financial status (χ^2^ = 17.389; *p* = 0.002), and self-assessment of general health (χ^2^ = 26.943; *p* < 0.001) with all examined levels of health literacy.

In addition, Table 4 shows that according to gender, males and females had similar mean values (29.60 ± 8.97 versus 28.68 ± 9.37) for the general health literacy index. Furthermore, younger respondents, up to the age of 50 years, showed better mean values for the health literacy index in comparison with those older than 65 (34.76 ± 9.45 versus 29.00 ± 9.37). Respondents with a primary (21.59 ± 7.06) and medium school education (26.68 ± 6.48) showed a lower health literacy index in comparison to those with a higher education (38.19 ± 5.69).

In addition, the value of the health literacy index was higher in employed respondents compared with unemployed respondents (34.43 ± 7.84 versus 28.71 ± 9.10). A better health literacy index was evident in respondents who perceived their financial status as good (34.27 ± 7.50 versus 29.99 ± 9.07), in people who perceived their general health as good (35.40 ± 7.03 versus 25.84 ± 8.92), in people with a normal BMI (30.90 ± 9.33 versus 28.96 ± 8.89), and in people with a normal systolic blood pressure (30.94 ± 8.97 versus 27.55 ± 9.08).

Significantly associated with all examined levels of health literacy were education level (χ^2^ = 110.426; *p* < 0.001), employment status (χ^2^ = 7.461; *p* = 0.024), self-assessment of financial status (χ^2^ = 17.389; *p* = 0.002), and self-assessment of general health (χ^2^ = 26.943; *p* < 0.001).

Significant associations with the general health literacy index were education level (*F* = 104.763; *p* < 0.001), employment status (*t* = 2.572; *p* = 0.011), self-assessment of financial status (*F* = 15.788; *p* < 0.001), self-assessment of general health (*F* = 24.562; *p* < 0.001), body mass index (*F* = 4.997; *p* = 0.008), and systolic blood pressure (*F* = 3.661; *p* < 0.027).

### 3.5. Association between Health Literacy and Quality of Life

We applied linear regression to investigate the connection between health literacy and various domains of patients’ quality of life, as shown in Table 5. We identified that health literacy was highly statistically significant (*p* < 0.001) and an independent predictor of quality of life (physical, mental, and total quality of life) in patients with heart failure, both before and after adjustment for sociodemographic characteristics (sex, age, marriage, education, employment status, financial status perception, number of visits to a doctor, smoking, and alcohol).

## 4. Discussion

This study was designed to assess health literacy and quality of life in heart failure patients, identify the factors associated with health literacy, and investigate the connection between health literacy and quality of life.

There are several studies that have investigated a correlation between health literacy and quality of life in patients with heart failure [9,12,13,14,15,16,17,18].

Furthermore, health literacy has not been studied in Serbian patients with heart failure, although in this country, heart failure is one of the 10 most common causes of death [32].

The results of our study revealed limited health literacy in just over half (64%) of our respondents. These results are very similar to a previous study in Taiwan, which was also conducted in an urban hospital involving inpatients diagnosed with NYHA II and III heart failure, and in which 60.2% of the respondents showed inadequate health literacy [12]. A previous study [25] conducted in Spain showed a higher level of limited health literacy (79.6%) in patients with heart failure in the primary health care sector.

According to a systematic literature review [8], previous studies of health literacy in patients with heart failure were mostly undertaken in the United States using the S-TOFHLA questionnaire and showed that, on average, 39% of patients (range: 17.5%–97%) had low levels of health literacy.

Previous studies have highlighted the need to use a comprehensive health literacy measuring instrument which can provide answers to investigations across all dimensions of health literacy [8]. In our study, we used the European Health Literacy Questionnaire, which enabled us to demonstrate the value of the health literacy index at different sublevels, and this specifically targeted the domains in which patients are least likely to cope [21].

Among the three explored dimensions, this examination showed that patients with heart failure had the lowest literacy in the area of disease prevention, which is consistent with the fact that heart failure can be prevented [6]. Furthermore, previous studies showed that the use of preventive services was diminished in people with reduced literacy [7].

Our respondents had the best health literacy skills in the health care dimension. One possible reason for this could be that patients are covered with compulsory insurance, with available access to general practitioners.

In the present study, the mean health literacy index was 29.23 ± 9.12, which represented a somewhat better health literacy than in the Spanish study (25.4 ± 9.1) [25]. Although low health literacy is associated with an increased number of hospitalizations related to heart failure [8], one possible reason as to why our inpatients had a somewhat better health literacy than outpatients was the conditions under which our patients were interviewed. For example, our patients completed their questionnaire on their last day of hospitalization in a stable state and in a quiet environment provided by a hospital room. Furthermore, our patients did not have any comorbidities, as such patients were excluded from our study.

Another aim of our study was to highlight the reports of both generic and specific questionnaires relating to quality of life, thus affording us the possibility of comparing studies involving the quality of life of cardiovascular patients [10,19,20]. This was particularly important because only a few previous studies have used two quality of life questionnaires to investigate patients with heart failure [33]. Along with a specific questionnaire, it is recommended that such studies use generic measuring instruments to evaluate quality of life in order to acquire a more objective picture of the patient’s life, not just disease-related problems [10,20].

Our current results, using two questionnaires to acquire data relating to quality of life, showed that our patients had a poorer quality of life in the physical dimension. This was very similar to a previous study [34] involving patients with heart failure in the primary health care sector in Spain, which also used the same questionnaires relating to quality of life. Furthermore, when considering individual dimensions of quality of life, as measured by the SF-36, the worst values in our study were related to the physical role and physical functioning, which has been proven in patients with heart failure compared to other serious chronic diseases [11]. These results are in accordance with the fact that patients with heart failure usually have a poor quality of life in the physical dimension due to the very nature of the disease [11]. Many previous studies [5,6,10,11,33] have shown that heart failure has a particularly strong effect upon quality of life.

The best scores for quality of life in our present study were identified in the emotional role and social functioning. This was also shown in a previous study [34], except that values relating to the emotional dimension of quality of life were somewhat better in our study. One possible reason for this could be that we excluded patients with psychiatric disorders, as well as those with impaired cognitive functions. Previous studies [10,29] have shown that depression is very common in people with heart failure.

A study carried out by Gonzales and colleagues highlighted a lack of research relating to the impact of educational level, demographic variables, and other sociodemographic characteristics on health literacy in cardiovascular patients [19].

The mean age of our respondents was 70.12 ± 9.63 years. Our patients were all over 18 years of age and 73.5% of our study population was older than 65 years of age. This is in accordance with the global literature [6], which shows that over 80% of patients with heart failure are older than 65 years. Our study showed that health literacy worsened with increasing age, although this was not statistically significant. The same finding has been reported in many previous studies of patients with heart failure [8].

Our respondents with a poorer health literacy had a statistically significantly lower level of education, were unemployed, and their financial status was assessed as poor, which is in line with other studies of patients with heart failure [8,9,12].

Self-perceived health in general has been rarely examined in other similar studies. However, our results showed that heart failure patients who perceived their health as being poor had a lower level of health literacy. Sorensen and colleagues also came to the same conclusion in their study [21].

In our study, BMI was statistically associated with health literacy index; this result was similar to that of previous studies of the European population [21]. We also found that increased systolic blood pressure was also significantly associated with the health literacy index; previous studies reported that this specific parameter was a significant predictor of mortality in patients with heart failure [5].

When we investigated the connection between health literacy and quality of life, the results showed that health literacy among our heart failure patients was highly statistically significant and was an independent predictor of quality of life, and its physical and mental dimensions, as well as the overall quality of life. This was the case both before and after adjustment for sociodemographic characteristics (sex, age, marriage, education status, visits to the doctor, smoking, and alcohol).

Previous articles [8,9,12] also showed that age, sex, level of education, and financial status represented independent predictors of low health literacy in patients with heart failure.

Our results demonstrated a correlation between health literacy and quality of life in patients with heart failure, as seen in previous studies [9]. In addition, our results were in agreement with previous studies of a European population that used the HLQ-EU-Q47 and SF-36 measuring instruments [23].

### Limitations

This study was conducted as a cross-sectional study and can only therefore be interpreted in relation to an urban environment in a city in Serbia. Furthermore, our investigations were limited to inpatients in the cardiology department of a specific hospital. We also used specific exclusion criteria, which may have had an impact on the emotional aspect of quality of life, as well as some of the other variables examined. Our exclusion criteria thus enabled us to select typical patients with heart failure without the presence and impact of severe comorbidities. We only selected NYHA II and III patients in the stable phase.

## 5. Conclusions

Our current analysis showed that the level of limited health literacy in hospitalized patients with heart failure was 64%. Health literacy was also shown to be a highly statistically significant and independent predictor of the quality of life of patients with heart failure. The value of the health literacy index was 29.23. Lower levels of education, unemployment, higher BMI, increased systolic blood pressure, poor financial status, and poor health were generally found to be significantly associated with health literacy.

## Figures and Tables

**Table 1:** Sociodemographic characteristics of the respondents in the study.

Sociodemographic Characteristic		No.	%
Total sample size		200	100
Gender	Male	119	59.5
	Female	81	40.5
Age	≤50	5	2.5
	51–64	48	24
	≥65	147	73.5
Marital status	Married	122	61
	Unmarried	78	39
Education	Low ≤ 8	47	23.5
	Medium 8–12	88	44
	High > 12	65	32.5
Employment	Employed	18	9
	Unemployed	182	91
Financial status perception	Poor	51	25.5
	Average	96	48
	Good	53	26.5
Self-perceived health	Poor	115	57
	Average	44	22
	Good	41	20.5
Number of visits to the doctor	1–4	173	86.5
	3 months ago	18	9
	Have not seen	9	4.5
Cigarettes	Yes	36	18
	No	164	82
Alcohol	Yes	18	9
	No	182	91
BMI	Kg/m^2^	28.17 ± 5.18	
Blood pressure	Systolic	126.83 ± 22.47	
	Diastolic	78.46 ± 14.20	

**Table 2:** Health literacy score of the respondents according to HLS-EU-Q47.

Question Number from HLS-EU-Q47	Dimensions from HLS-EU-Q47	Mean, SD ^a^	Categorized Level of Health Literacy	Value of Health Literacy Score (%)
Q1–47	GENERAL HEALTH LITERACY INDEX	29.23 ± 9.12		
			1. Excellent > 42–50	16 (8%)
			2. Sufficient > 33–42	56 (28%)
			3. Problematic > 25–33	55 (27.5%)
			4. Inadequate 0–25	73 (36.5%)
			5. Limited (3 + 4) 0–33	128 (64%)
Q1–16	HEALTH CARE	33.03 ± 8.52		
			1. Excellent > 42–50	31 (15.5%)
			2. Sufficient > 33–42	74 (37%)
			3. Problematic > 25–33	54 (27%)
			4. Inadequate 0–25	41 (20.5%)
			5. Limited (3 + 4) 0–33	95 (47.5%)
Q17–31	PREVENTION OF DISEASE	28.01 ± 9.34		
			1. Excellent > 42–50	17 (8.5%)
			2. Sufficient > 33–42	43 (21.5%)
			3. Problematic > 25–33	66 (33%)
			4. Inadequate 0–25	74 (37%)
			5. Limited (3 + 4) 0–33	140 (70%)
Q32–47	HEALTH PROMOTION	30.46 ± 9.69		
			1. Excellent > 42–50	24 (12)
			2. Sufficient > 33–42	62 (31%)
			3. Problematic > 25–33	44 (22%)
			4. Inadequate 0–25	70 (35%)
			5. Limited (3 + 4) 0–33	114 (57%)

^a^ Mean of score of the general health literacy index and three subindices. Score of the general health literacy index was 50, while the smallest value was zero.

**Table 3:** Scores on quality of life from SF-36 and MLWHF.

Quality of Life Questionnaire	Dimensions of Quality of Life	Mean (SD)	Minimum Score	Maximum Score	Median (Range)
SF-36 *	Physical functioning	38.77 (30.64)	0	100	32.50 (58.75)
	Physical role	33.12 (41.63)	0	100	0 (75.00)
	Body pain	61.99 (35.54)	0	100	62.00 (69.00)
	General health	49.57 (24.45)	0	100	45.00 (40.00)
	Vitality	42.37 (28.27)	0	90	35.00 (53.75)
	Social functioning	50.26 (30.44)	0	100	50.00 (50.00)
	Emotional role	50.50 (46.80)	0	100	67.00 (100.00)
	Mental health	59.04 (25.25)	4	100	60.00 (40.00)
	Total physical dimensions	45.01 (24.77)	0	97	40.00 (39.50)
	Total mental dimensions	50.35 (25.82)	1	97	49.00 (47.50)
Minnesota **	Physical dimension	24.73 (10.87)	1	40	26.00 (19.50)
	Emotional dimension	7.34 (6.42)	0	25	6.00 (25.00)
	Total quality of life	51.67 (24.10)	4	98	50.00 (40.00)

* SF-36: Higher values speak in favour of a better quality of life; ** Minnesota: Lower values speak in favour of a better quality of life.

**Table 4:** Respondents’ sociodemographic characteristics by health literacy levels.

Sociodemographic Characteristics			Level of Health Literacy			*p* *	Mean (SD) General Health Literacy Index	*p* **
			Limited (0–33), %	Sufficient (> 33–42), %	Excellent (> 42), %			
Gender	Male		74 (62.2%)	37 (31.1%)	8 (6.7%)	0.416	29.60 (8.97)	0.485
	Female		54 (66.7%)	19 (23.5%)	8 (9.9%)		28.68 (9.37)	
Age (years)	≤50		2 (40%)	2 (40%)	1 (20%)	0.361	34.76 (9.45)	0.382
	51–64		32 (66.7%)	15 (31.3%)	1 (2.1%)		29.34 (8.28)	
	≥65		94 (63.9%)	39 (26.5%)	14 (9.5%)		29.00 (9.37)	
Marital status	Married		73 (59.8%)	39 (32%)	10 (8.2%)	0.270	29.93 (9.14)	0.174
	Unmarried		55 (70.5%)	17 (21.8%)	6 (7.7%)		28.13 (9.04)	
Education	Low ≤8		45 (95.7%)	1 (2.1%)	1 (2.1%)	<0.001	21.59 (7.06)	<0.001
	Medium 8–12		74 (84.1%)	14 (15.9%)	0 (0%)		26.68 (6.48)	
	High >12		9 (13.8%)	41 (63.1%)	15 (23.1%)		38.19 (5.69)	
Employment	Employed		7 (38.9%)	10 (55.6%)	1 (5.6%)	0.024	34.43 (7.84)	0.011
	Unemployed		121 (64%)	46 (25.3%)	15 (8.2%)		28.71 (9.10)	
Financial status perception	Poor		40 (78.4%)	10 (19.6%)	1 (2%)	0.002	29.99 (9.07)	<0.001
	Average		65 (67.7%)	25 (26%)	6 (6.3%)		28.69 (8.72)	
	Good		23 (43.4%)	21 (39.6%)	9 (17%)		34.27 (7.50)	
Self-perceived health	Poor		90 (78.3%)	20 (17.4%)	5 (4.3%)	< 0.001	25.84 (8.92)	< 0.001
	Average		23 (52.3%)	17 (38.6%)	4 (9.1)		32.32 (7.16)	
	Good		15 (36.6%)	19 (46.3%)	7 (17.1)		35.40 (7.03)	
Number of visits to the doctor	1–4		109 (63%)	51 (29.5%)	13 (7.5%)	0.454	29.43 (8.93)	0.370
	3 months ago		14 (77.8%)	2 (11.1%)	2 (11.1)		26.49 (9.8)	
	Have not seen		5 (55.6%)	3 (33.3%)	1 (11.1%)		30.89 (11.41)	
Cigarettes	Yes		24 (66.7%)	11 (30.6%)	1 (2.8%)	0.439	28.41 (9.34)	0.555
	No		104 (63.4%)	45 (27.4%)	15 (9.1%)		29.41 (9.09)	
Alcohol	Yes		15 (83.3%)	3 (16.7%)	0 (0%)	0.162	25.15 (8.29)	0.046
	No		113 (62.1%)	53 (29.1%)	16 (8.8%)		29.63 (9.1)	
BMI	< 18.5 Malnourished		4 (100%)	0 (0%)	0 (0%)	0.313	16.47 (4.54)	0.008
	18.5–24.9 Normal weight		26 (52%)	18 (36%)	6 (12%)		30.90 (9.33)	
	> 25 Obese		95 (66.9%)	37 (26.1%)	10 (7%)		28.96 (8.89)	
Blood pressure	Systolic	Low	3 (75%)	1 (25%)	0 (0%)	0.153	26.58 (7.44)	0.027
		Normal	57 (57%)	33 (33%)	10 (10%)		30.94 (8.97)	
		Elevated	68 (70.8%)	22 (22.9%)	6 (6.3%)		27.55 (9.08)	
	Diastolic	Low	2 (66.7%)	1 (33.3%)	0 (0%)	0.583	22.73 (13.89)	0.127
		Normal	53 (60.9%)	25 (28.7%)	9 (10.3%)		30.50 (8.63)	
		Elevated	73 (66.4%)	30 (27.3%)	7 (6.4%)		28.40 (0.30)	

* χ^2^ test; ** *t* test and ANOVA.

**Table 5:** Association between health literacy and quality of life.

Quality of Life Questionnaires		Health Literacy					
		β Unadjusted	Coefficient (95% CI)	*p*	β Adjusted	Coefficient (95% CI)	*p*
SF-36	Physical dimension ^a^	0.534	1.13; 1.77	<0.001	0.42	0.71; 1.57	<0.001
	Emotional dimension ^a^	0.513	1.11; 1.79	<0.001	0.38	0.62; 1.55	<0.001
Minnesota	Total quality of life ^a^	-0.417	-1.44; -0.76	<0.001	-0.30	-1.25; -0.33	0.001

^а^ Adjusted to gender, age, marriage, education, employment status, self-assessment of financial status, number of visits to the doctor, smoking, and alcohol.
